# Preoperative radiological compression features and their relationship with pre- and postoperative visual field defects in pituitary macroadenomas: a retrospective cohort from the neuro-ophthalmological clinic

**DOI:** 10.3389/fopht.2026.1724655

**Published:** 2026-04-10

**Authors:** Lorna Zwirs-Grech Fonk, Marjolein Tabak, Fleur I. Notting, Berit M. Verbist, Amir H. Zamanipoor Najafabadi, Luc van Vught, Gregorius P. M. Luyten, Marco J. T. Verstegen, Stijn W. van der Meeren, Nienke R. Biermasz, Jan-Willem M. Beenakker, Irene C. Notting, Iris C. M. Pelsma

**Affiliations:** 1 Department of Ophthalmology, Leiden University Medical Center, Leiden, Netherlands; 2 Department of Radiology, Leiden University Medical Center, Leiden, Netherlands; 3 Department of Neurosurgery, Leiden University Medical Center, Leiden, Netherlands; 4 Department of Internal Medicine, Division of Endocrinology, Leiden University Medical Center, Leiden, Netherlands; 5 Department of Radiation Oncology, Leiden University Medical Center, Leiden, Netherlands

**Keywords:** Fujimoto, Humphrey Field Analyser, MRI, PitNET, pituitary adenoma, radiological outcomes, visual field defects

## Abstract

**Purpose:**

The study aimed to correlate preoperative magnetic resonance imaging (MRI) characteristics with pre- and postoperative visual field deficits (VFDs) in patients with non-functioning pituitary macroadenoma (NFMA).

**Methods:**

This retrospective cohort study included 74 patients with preoperative high-quality MRI sequences and detailed visual field assessments (including masked ophthalmologist interpretation of perimetry) before and <9 months postoperatively. Patients (median age 59 years; 43% female) underwent surgery between 2005 and 2016. Two neuro-ophthalmologists independently scored visual fields (i.e., affected, pattern, improvement) while masked to mean deviations (MD). Radiological contact, deformity, hyperintensity, and atrophy of the optic system (nerves, chiasm [Fujimoto grading], and tracts) were scored. Generalized estimating equations (GEE) were used for correlation analyses. Receiver operating characteristic (ROC) analysis was used to determine MD cutoff values.

**Results:**

Higher preoperative Fujimoto grades correlated with lower preoperative MD (P <0.001). Hyperintensity of the optic system was observed in five of 74 patients. Temporal hemianopia and quadrantanopia (47%) were the most frequently observed preoperative VFD patterns, even in the eyes with optic nerve or tract compression. A preoperative MD cutoff of −1.41 dB (ROC sensitivity 92%, specificity 95%) was suggested for determining affected visual fields. Moreover, an MD cutoff Δ2.52 dB (ROC sensitivity 87%, specificity 78%) was suggested for improvement of visual fields.

**Conclusions:**

Preoperative optic chiasm compression results in lower MD values. Compression of the optic nerve or tract was not associated with postoperative MD in this patient population. Following the ROC analysis, we suggest using MD ≤−1.41 dB to determine preoperatively affected visual fields for research purposes.

## Introduction

Pituitary adenomas account for approximately 15% of diagnosed intracranial neoplasms. Compression of the optic chiasm and surrounding structures may lead to visual field defects (VFDs; 46%–75% of patients) and decreased visual acuity (14%–44% of patients) (1–3).

Endoscopic endonasal transsphenoidal surgery is an effective technique for adenoma resection aimed at improving vision (4–6). VFDs and decreased visual acuity improve or normalize after surgery in most patients, whereas a small subset have unchanged or worsened VFDs (7–9). Prediction of VFD outcome, as well as the pathophysiological mechanisms underlying the deterioration of visual function after chiasmic decompression, is not well understood (10). Increased insight into these mechanisms may aid a more reliable prognosis of VFD recovery, as well as improved timing of surgery for individual patients.

Magnetic resonance imaging (MRI) is the most widely used technique for structural examination of the pituitary and sellar region (11, 12). The severity of optic chiasm compression by the tumor (categorized by Fujimoto grade (13)), including coronal and sagittal displacement of the optic chiasm, influences preoperative VFDs (14, 15). Moreover, worse preoperative VFDs have been linked to a reduced chance of complete VFD recovery (16–23). Other predicting factors for visual outcome have been reported, e.g., apoplexy, tumor size (18, 19), suprasellar extension of the tumor mass (24, 25), and the degree of optic atrophy (19).

To date, detailed radiological assessment, including the severity and location of optic system compression, has not been studied or implemented in clinical care. The aim of this study is therefore to investigate the relationship between detailed radiological anatomical assessment and detailed pre- and postoperative visual fields, ultimately aiming to correlate specific locations of compression with the visual field patterns by assessing preoperative MRI characteristics and detailed pre- and postoperative visual field deficits (VFDs).

## Methods

### Ethics

This study was approved by the Scientific Committee of the Department of Internal Medicine at Leiden University Medical Center (LUMC; G19.011), and the need for informed consent was waived.

### Patient population

The LUMC is a tertiary referral center for pituitary tumors and anterior skull base pathology in the Netherlands. Patients are assessed by a multidisciplinary team consisting of endocrinologists, neurosurgeons, ophthalmologists, and radiologists to decide on the surgical indication and its urgency.

For the present study, all patients who underwent surgery in our center between 2003 and 2017 (either as monotherapy or as combination therapy with pharmacological treatment or radiotherapy) were retrospectively identified. Patients were included when (a) age at surgery was ≥18 years, (b) nonfunctioning macroadenoma (NFMA) was diagnosed clinically and radiologically, (c) an expert ophthalmologist evaluation was indicated and performed, including visual field assessment using the Humphrey Field Analyzer (HFA), before surgery and within <9 months after surgery, and (d) high-quality MRI sequences were present. Patients were excluded if visual field (VF) assessments were unreliable (*vide infra*), or if other ocular or neurological comorbid pathologies potentially leading to VFDs (e.g., glaucoma, multiple sclerosis, retinal artery occlusion) were present.

**Figure 1:**
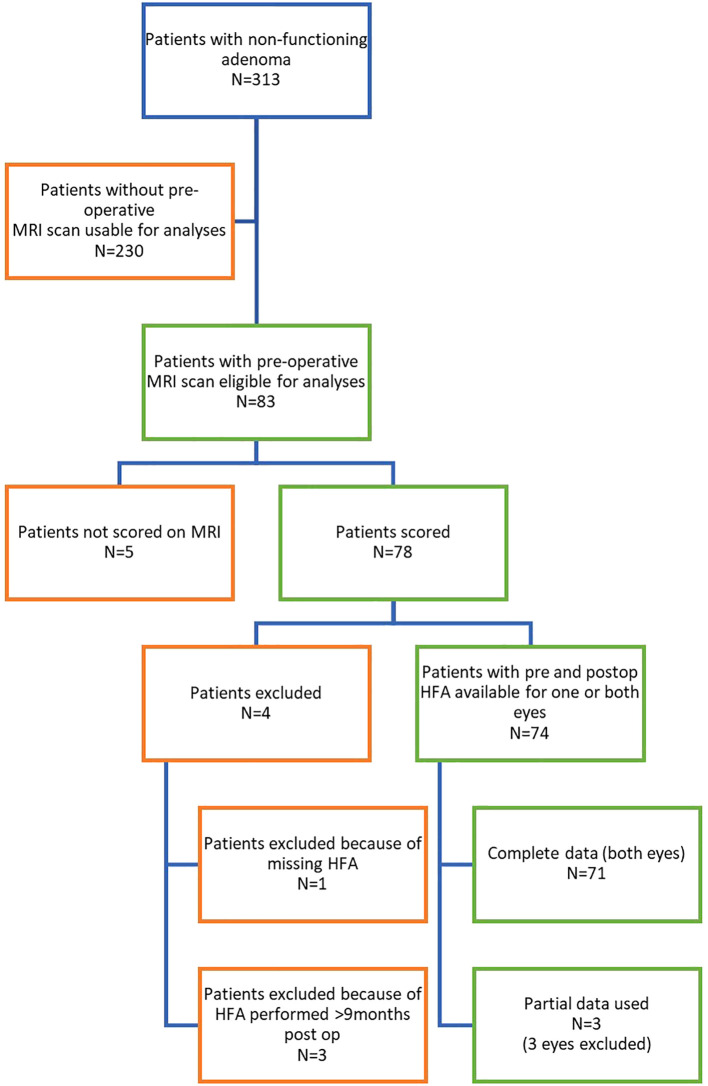
Flowchart of inclusion. From 313 patients with NFMAs operated between 2003-2017, preoperative MRI scans with all sequences/contrast were not available for 230 patients. Of the 83 patients with eligible MRI scans, five patients were excluded due to MRI scoring difficulties, and four were excluded due to insufficient ophthalmological data. Ultimately, 74 patients were included in this study (both eyes in 71 patients, and one eye in three patients), who underwent surgery between 2005 and 2016. HFA, Humphrey Field Analyzer; MRI, magnetic resonance imaging; N, number of patients; n, number of eyes.

From the larger surgically treated cohort of 313 patients with NFMAs, 74 patients were included in this study (both eyes in 71 patients and one eye in three patients), as shown in **Figure 1**.

### Study parameters

For included patients, clinical characteristics were extracted from the electronic patient file, e.g., sex, age, tumor type, tumor size, presence of apoplexy, type of treatment modality, and comorbidities.

### Preoperative radiological measures

#### Magnetic resonance imaging

Imaging of the pituitary region was performed using MRI on a clinical scanner (≥1.5 Tesla, Philips Healthcare, Best, Netherlands). Images were acquired with and without gadolinium-based contrast (sagittal T1-weighted and coronal T1- and T2-weighted). More recently, the following acquisition parameters were used: coronal T2TSE (TR 2000, TE 90, FOV 120 × 122, slice thickness 2, acquisition matrix 300 × 272, acquisition voxel 0.40 × 0.45 × 2, reconstructed voxel 0.19 × 0.19 × 2), coronal t1 and contrast-enhanced coronal and sagittal T1TSE (TR 550, TE 17, FOV 120 × 120, slice thickness 2, acquisition matrix 200 × 200, acquisition voxel 0.6 × 0.6 × 2, reconstructed voxel 0.3 × 0.3 × 2). For standard-of-care purposes, MRI scans were reviewed and reported in the electronic patient file.

For the present study, an in-depth, structured reanalysis of the preoperative MRI scans was performed by a dedicated pituitary neuroradiologist (BV) to assess the impact of the optic system based on radiological imaging. The outcomes outlined below were recorded and analyzed.

#### Chiasm

**Figure 2:**
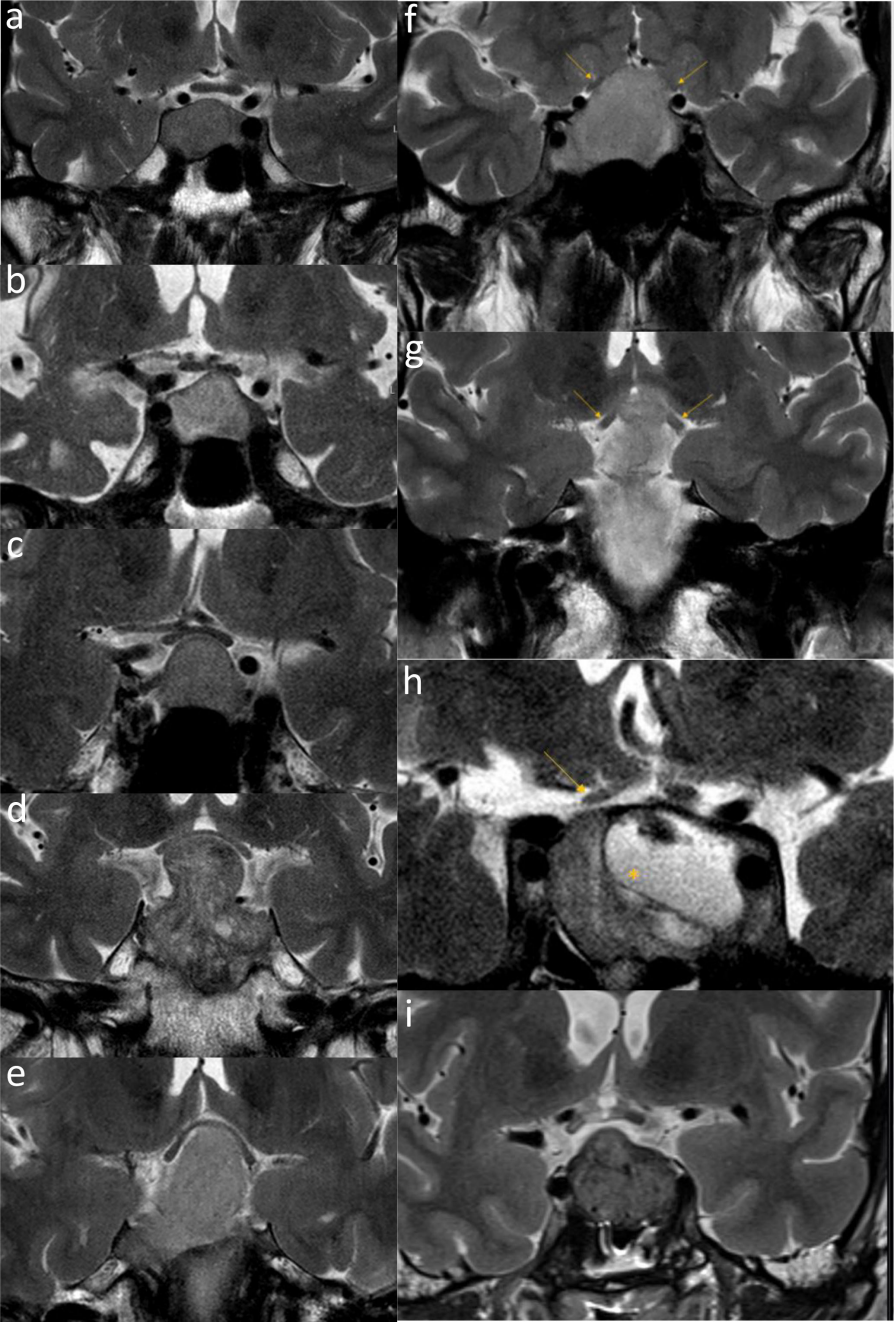
Coronal T2 weighted images showing different grades of compression on the optic system and hyperintensity of the optic nerve. Examples of the radiological scoring used in the present study are shown. Notably, all panels show radiological examination of different patients performed at different points in time with different MRI settings. **(A–E)** The five Fujimoto classification grades are: 0, tumor has no contact with optic chiasm; 1, tumor has contact with optic chiasm without deformity of the upper surface of optic chiasm; 2, tumor compresses the optic chiasm and produced the deformity of the upper surface and visible supra-chiasmal cistern; 3, tumor compresses the optic chiasm with invisible supra-chiasmal cistern; 4, tumor compresses the optic chiasm with cerebral deformity. **(F)** Compression of the prechiasmatic optic nerve: the adenoma has contact with the left optic nerve without any deformation (grade 1) and compresses and deforms the right optic nerve (grade 2). **(G)** Contact with the optic tract: the adenoma extends to the left optic tract (grade 1) while there is no contact with the right optic tract (grade 0). **(H)** Atrophy and increased signal intensity of the right optic nerve (arrow) in a patient with a mixed solid-cystic macroadenoma (asterisk) reaching up to the chiasm **(I)** Increased signal intensity without atrophy in the right optic tract.

First, the severity of optic chiasm compression was classified using the criteria of Fujimoto et al. (13), for which compression could range from grade 0 (no contact) to grade 4 (contact and deformity of the chiasm and cerebral structures). Examples of Fujimoto scoring in the included patients are shown in **Figures 2A–E**.

#### Optic nerve and tract

As exemplified in **Figures 2F, G**, the severity of anatomical compression at the level of the optic nerves and optic tracts (left and right) was graded from 0 to 2, with grade 0 representing no contact of the NFMA with the nerve/tract, grade 1 representing contact of the NFMA with the nerve/tract without anatomical deformity, and grade 2 representing contact between the nerve/tract and NFMA resulting in anatomical deformity, using an institutional protocol.

#### Hyperintensity

Finally, the optic system was investigated for the presence of hyperintensity and/or atrophy on T2-weighted images, as shown in **Figures 2H, I**. The presence of (focal) hyperintensity and nerve atrophy was graded from 0 to 2, with 0 reflecting “no hyperintensity and no atrophy,” 1 “hyperintensity without atrophy,” and 2 “hyperintensity with atrophy.”

### Pre- and postoperative ophthalmological assessment

VF assessment was performed by static perimetry on an HFA (Zeiss Humphrey Systems, Dublin, California, USA), using the SITA Fast 30–2 threshold program (26). The main outcome of VF assessment was the mean deviation (MD) in decibels (dB) per eye, automatically corrected for age and gender. Notably, visual field index (VFI) was not used as an outcome measure because of its lower sensitivity and unavailability in the included cohort. The HFA is the most commonly used perimeter in patients with chiasm compression (27). As the HFA 24–2 is restricted on the temporal side to 24°, the HFA 30–2 program, assessing the temporal 30°, was favored (28). Perimetry was combined with a thorough ophthalmological evaluation to identify interfering factors (e.g., cataract). Eyes with unreliable VF measurements based on fixation loss (>3 losses), false positives (>10%), or false negatives (>15%) were excluded.

For quantitative analyses of MD values, automatic readout of digitized MD values was performed using an in-house-developed visual-recognition-based algorithm, an updated and publicly released version of the open-source VFPy toolbox (https://github.com/MREYE-LUMC/VFPy), as described by van Rozendal et al. (29). All digitized Humphrey Field Analyzer printouts were manually checked. The distribution at each test point was evaluated. To analyze the total peripheral visual field, the mean of each individual Humphrey Field Analyzer test point was compared between groups.

### Reliability analyses of visual field defects of expert opinion

Qualitative assessment of the pre- and postoperative visual fields was performed by two neuro-ophthalmologists (ICN and SvdM). Pre- and postoperative HFA assessments for both eyes, including the total deviation and pattern standard deviation (PSD) plots, were visually evaluated after removal of the numerical MD, PSD, and VFI scores. The experts independently scored whether the VFs were affected preoperatively (yes/no/unreliable/unavailable), the pattern of VF loss (e.g., temporal hemianopia, total anopia, artifacts), and whether the VF had improved, remained stable, or worsened after surgery. In cases of disagreement in scoring, the corresponding VFs were discussed with the two scorers and one independent researcher (MT). The consensus scores were used for further analyses.

### Statistical analysis

All statistical analyses were performed using SPSS Statistics v.29. Following normality testing, data were summarized as frequencies (number and percentage (%)), mean ± standard deviation (SD), or median with interquartile range (IQR). An intraclass correlation coefficient (ICC) was calculated for the scoring of the two experts using a two-way random-effects model with absolute agreement. Comparative analyses of both pre- and postoperative MD with radiological grading outcomes (e.g., Fujimoto grade) were performed using a generalized estimating equations (GEE) model, accounting for the linkage between right and left eyes and differences in follow-up duration between patients. For both the pre- and postoperative MD, differences between affected and unaffected eyes according to expert scoring were assessed using Mann–Whitney U tests. Receiver operating characteristic (ROC) analysis was performed to assess the most sensitive cutoff value for MD regarding affected and improved visual fields. To enable analysis of optic tract compression, MD outcomes were duplicated. The MD of one eye was linked with compression of the right and left optic tracts. P values <0.05 were considered significant.

## Results

### Clinical characteristics of the study population

Clinical characteristics are reported in **Table 1**. The median age of included patients was 59 years (IQR 44–68), and 32 (43%) patients were female. Ten patients (13%) had undergone prior surgery. The median follow-up duration was 3.0 months (IQR 1.47–4.7).

**Table 1:** Clinical characteristics of the patient population.

Baseline characteristics		All patients N = 74
Age (years)		59 (IQR 44–67)
Sex (female)		32 (43.2%)
Hypopituitarism		55 (74%)
No previous treatment		63 (85.1%)
Previous treatment modality	Surgery	9 (12.2%)
	Surgery + RT	1 (1.4%)
	PharmaT	1 (1.4%)
Current preoperative tumor size	Macroadenomas	63 (85.1%)
	Giant adenomas	2 (2.7%)
	Macro remnant	8 (10.8%)
	Giant remnant	1 (1.4%)
Apoplexy		1 (1.4%)
Preoperative MD	OD*	−4,3 (IQR −10,1 to −2,0)
	OS**	−5,1 (IQR −11,9 to −1,6)
Follow-up duration (months)		3.1 (IQR 1.4–4.7)

Clinical characteristics of the included 74 patients are described. Follow-up duration ranged from 0.1 to 8.5 months. Values are reported as N (%), or median (interquartile range, IQR), unless otherwise specified. * Available for 72, and ** available for 73 eyes, respectively.

N, number of patients; PharmaT, pharmacological therapy; RT, radiotherapy.

### Preoperative radiological scoring

Based on the 148 eyes assessed, the adenoma was in contact with the optic nerve in 15/148 cases and in contact with resulting deformity of the surrounding structures in 30/148 cases. In the remaining 103/148 cases, there was no contact with the optic nerve, as summarized in **Table 2**.

**Table 2:** Preoperative radiological scoring.

Radiological characteristics	Anatomical location	Grade	Right	Center/chiasm	Left
Compression and deformity N = 74	Prechiasmal optic nerve	No contact or deformity	51		52
		Contact	9		6
		Contact and deformity	14		16
	Optic chiasm (Fujimoto)	Grade 0 No contact		4	
		Grade 1 Contact with chiasm without deformity		8	
		Grade 2 Contact with chiasm with deformity		20	
		Grade 3 Invisible supra chiasmal cistern		17	
		Grade 4 Cerebral deformity		25	
	Optic tract	No contact or deformity	65		63
		Contact	3		5
		Contact and deformity	6		6
Hyperintensity and atrophy N = 62	Prechiasmal optic nerve	No hyperintensity or atrophy	59		60
		Hyperintensity without atrophy	1		0
		Hyperintensity with atrophy	1		2
	Optic chiasm	No hyperintensity or atrophy		61	
		Hyperintensity without atrophy		0	
		Hyperintensity with atrophy		1	
	Optic tract	No hyperintensity or atrophy	60		61
		Hyperintensity without atrophy	1		1
		Hyperintensity with atrophy	0		0

Outcomes of detailed preoperative MR assessments are summarized. For examples of radiological scoring, see **Figure 1**. Values are reported as N.

Compression on the optic chiasm (N = 74) was characterized using the Fujimoto score, resulting in four patients with Fujimoto grade 0, eight with grade 1, 20 with grade 2, 17 with grade 3, and 25 with grade 4.

Of the 74 patients with both optic tracts analyzed (n = 148), the adenoma was in contact with the optic tract in 8/148 cases and in contact with resulting deformity of the surrounding structures in 12/148 cases. Moreover, no contact or deformity of the tract was observed in 128/148 cases. Notably, all patients with optic tract compression had Fujimoto grade 4.

Hyperintensity of the optic system could be assessed in 62 patients (124 eyes), as shown in **Table 2**. Of the five patients with hyperintensity of the optic nerve, chiasm, or tract, one had hyperintensity and atrophy of the optic nerve, optic chiasm, and tract and a complete visual field defect. One patient with optic nerve hyperintensity and atrophy showed deterioration (MD −26.69 dB to 28.14 dB). Another showed improvement (−5.7 dB to −3.51 dB). One patient with hyperintensity without atrophy showed slight improvement (−5.87 dB to −5.39 dB), and one patient with optic tract hyperintensity improved to completely normal visual fields. No patients showed hyperintensity on both optic nerves/tracts. .

### Ophthalmological assessment

**Figure 3:**
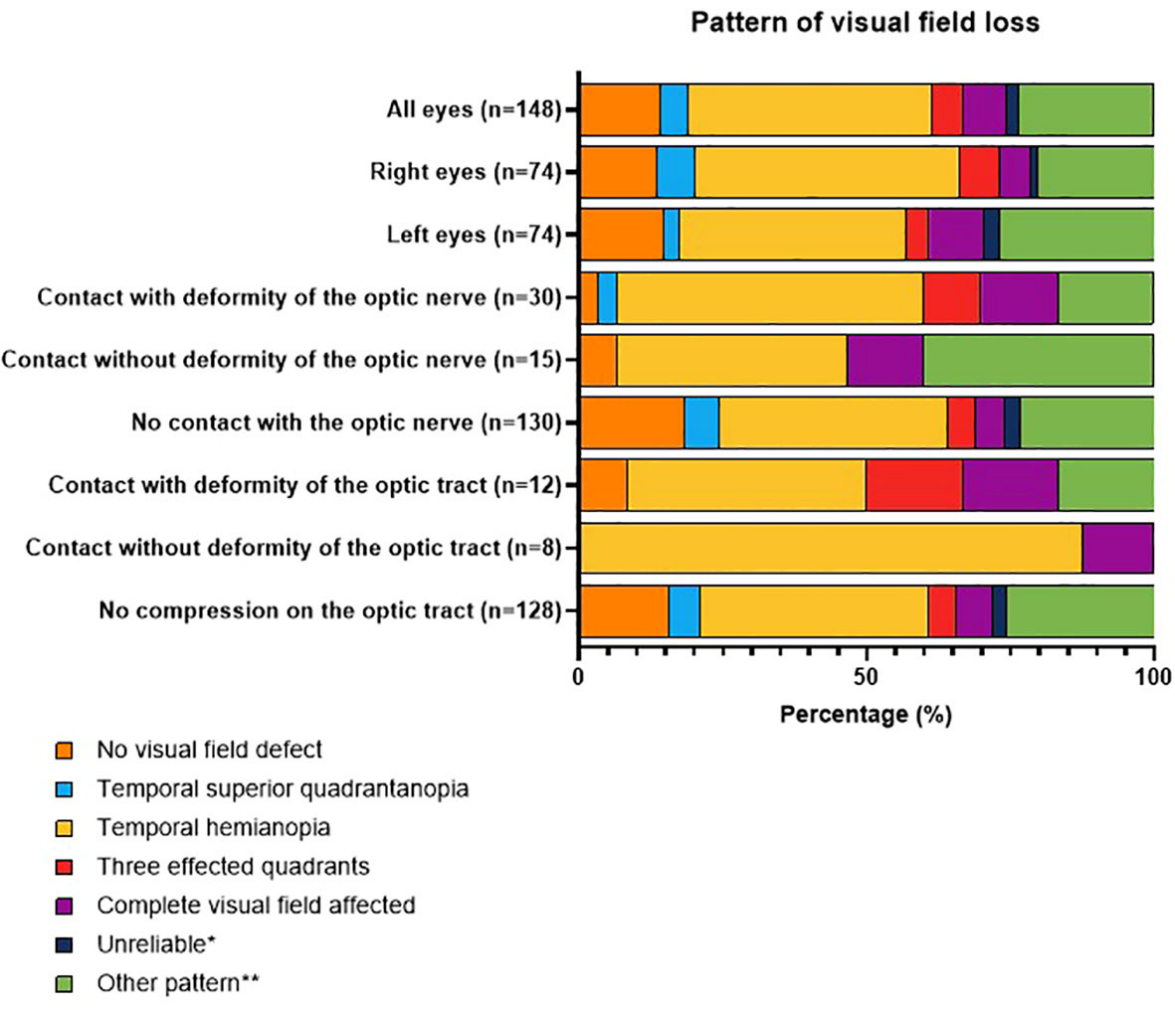
Patterns of visual field loss. Distribution of pattern of visual field loss categorized by severity and location of compression.

Expert reproducibility scores of pre- and postoperative VFD and the pattern of preoperative VFD were high, with ICC scores of 0.821–0.926 (*P*<0.0001), as summarized in **Table 3**. Of the 72 right eyes (OD), 62 (86%) were considered to have VFD, and 10 (13%) were unaffected. Of the 73 left eyes (OS), 62 (84%) were considered to have VFD, and 11 (15%) were unaffected. As shown in **Figure 3**, the most frequently observed pattern of VFD was temporal hemianopia (OD: 34/62 (55%), OS: 29/62 (47%)). Other non-classical forms of VFD were present in 15/62 (24%) right eyes and 19/62 (31%) left eyes. Patients with preoperative VFD, as classified by our expert panel, had significantly lower MD compared with the group without VFD (OD: −5.14 dB (IQR −11.4 to −2.9) vs. −0.7 dB (IQR −0.8–.3), *P* < 0.001 Mann–Whitney U test; OS: −8.1 dB (IQR −12.8 to −3.3) vs. −0.4 dB (IQR −0.9–0.5), *P* < 0.001).

**Table 3:** Reliability analyses of expert opinion scoring.

Expert opinion scoring	OD		OS	
	ICC (95%CI)	P-value	ICC (95%CI)	P-value
Preoperative visual field loss	0.894 (0.826–0.934)	<0.0001	0.926 (0.882–0.953)	<0.0001
Pattern of preoperative visual field loss	0.860 (0.778–0.912)	<0.0001	0.845 (0.745–0.903)	<0.0001
Postoperative improvement of visual field loss	0.821 (0.715–0.887)	<0.0001	0.900 (0.851–0.937)	<0.0001

Blinded assessment of the VFDs was carried out by two neuro-ophthalmologists (ICN and SvdM) for reliability analysis. The experts independently scored whether the VFs were affected preoperatively, and what type of pattern of VF loss (e.g., temporal hemianopia or total anopia) was observed. Reproducibility of the expert opinion was assessed by the intra-class correlation coefficient (ICC; 95% confidence interval) using two-way mixed models for single measurements.

Postoperative VFD were improved in 45/62 right eyes and 51/62 left eyes. Median preoperative MD values improved significantly following surgery from preoperative MD −4.67 dB (IQR −10.92 to −1.87) to −1.46 dB (IQR −3.27 to −0.30) (*P* < 0.001).

**Figure 4:**
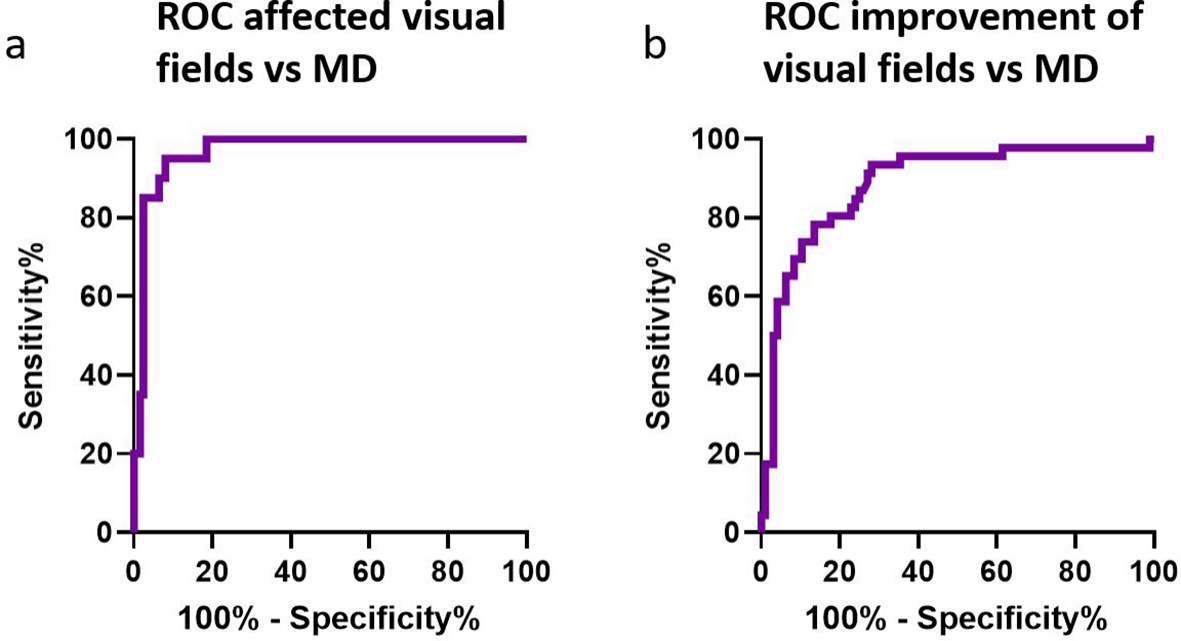
ROC for affected visual fields and MD improvement. **(A)** ROC curve showing whether preoperative visual fields were affected according to experts vs. MD. **(B)** ROC curve showing whether the postoperative visuals fields were improved compared to the preoperative visual fields according to experts vs. MD. MD, mean deviation; ROC, receiver operator curve.

The ROC curve shown in **Figures 4A**, comparing expert opinion (affected vs. not affected) with the MD score, resulted in an AUC of 0.968 (95% CI 0.940–0.996). The optimal cutoff value was −1.41 dB, with a sensitivity of 92% and specificity of 95%. Other cutoff values were −0.97 dB (sensitivity 98%, specificity 86%) and −2.69 dB (sensitivity 82%, specificity 100%).

The ROC curve shown in **Figures 4B**, comparing expert opinion on improvement with the ΔMD after surgery, resulted in an AUC of 0.887 (95% CI 0.825–0.949). The optimal cutoff value was Δ2.52 dB (sensitivity 72%, specificity 94%). Another cutoff value was Δ1.24 dB (sensitivity 87%, specificity 78%).

### Relationship between ophthalmological assessment and radiological scoring

The pattern of VFD, as determined by the expert evaluation, and the prevalence compression/deformity of the optic nerve are shown in **Figure 3**. The different patterns of visual field loss occurred in all patient groups and did not show a clear relationship with adenoma contact with the optic tract or nerve *per se*. Notably, no patients with optic tract compression had nasal hemianopia, and although patients with optic nerve contact without deformity more often showed an “other” pattern, patients with contact and deformity did not.

### Relationship between MD and radiological scoring

**Figure 5:**
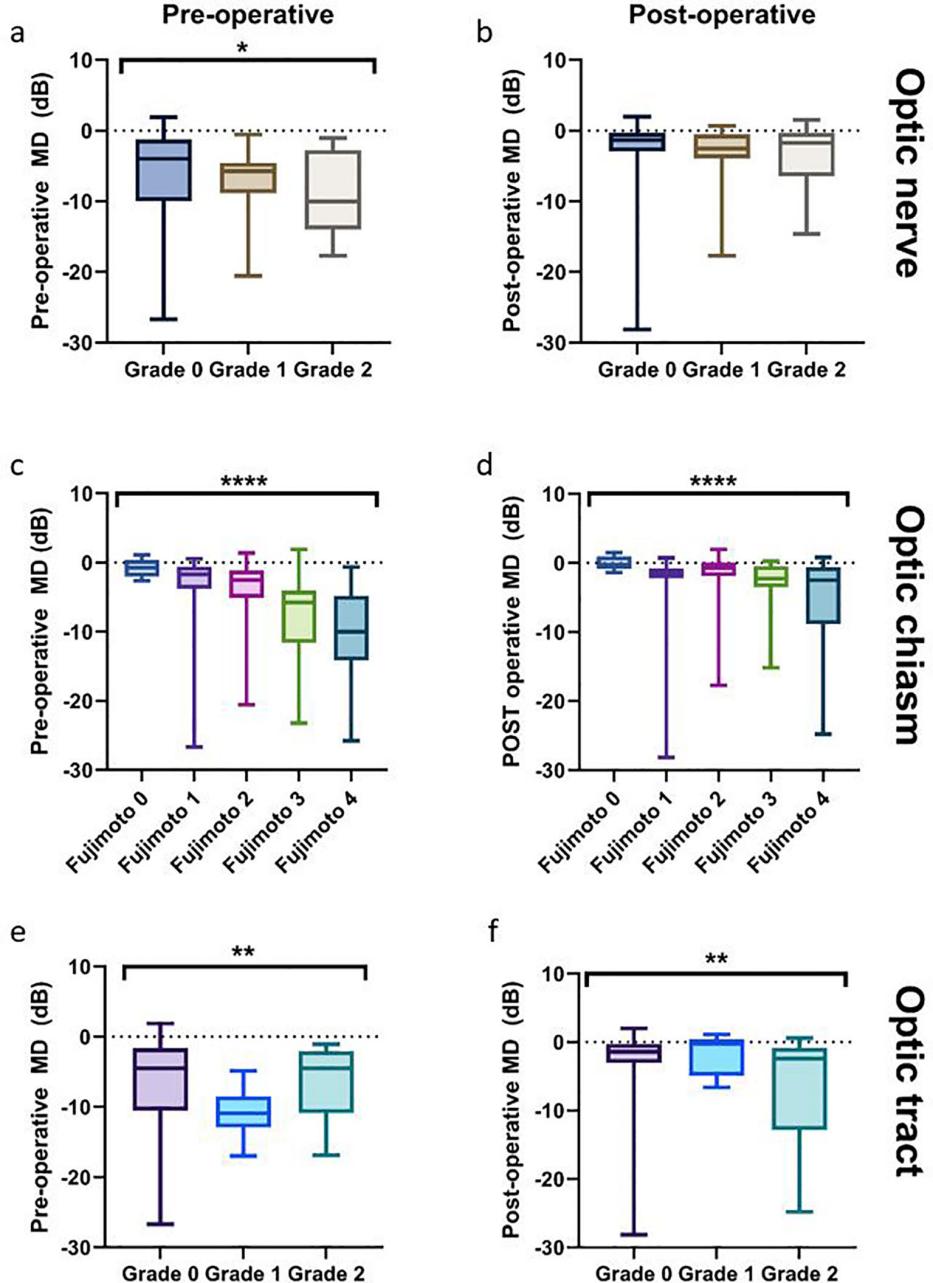
Boxplots with MD score before and after surgery by location and severity of compression. **(a, b)** MD score for severity of compression on the optic nerve before and after surgery. **(c, d)** MD score for severity of compression on the optic chiasm (Fujimoto score) before and after surgery. **(e, f)** MD score for severity of compression on the optic tract before and after surgery. *P <0.05, **P <0.01, ***P <0.005, ****P <0.001.

Eyes with contact and deformity of the optic nerve had lower preoperative MD compared with eyes without contact with the optic nerve (−10.03 (IQR −16.15 to −2.76) vs. −3.98 (IQR −9.98 to −1.23), P = 0.043, GEE model). Postoperative MD and optic nerve contact was not correlated (P = 0.407), as shown in **Figures 5A, B**.

Higher Fujimoto grades were correlated with lower preoperative MD, independent of left and right eyes. Preoperative MD values were significantly different between all Fujimoto groups (P <0.001, **Figure 5C**), with the exception of grade 0 vs. grade 1, grade 0 vs. grade 2, grade 1 vs. grade 2, and grade 3 vs. grade 4. Postoperative MD scores were significantly lower for Fujimoto grades 1, 3, and 4 vs. grade 0 (P = 0.043, P = 0.009, P = 0.002, respectively), and for grade 2 vs. grade 3 (P = 0.018), and grade 2 vs. grade 4 (P <0.001) (**Figure 5D**).

Despite the small number of patients exhibiting optic tract compression, significantly lower preoperative MD was observed in patients with optic tract contact without deformity compared with the group without contact (*P* = 0.003). However, preoperative MD was not significantly different in patients with contact and deformity of the optic tract (*P* = 0.39, **Figure 5E**). By contrast, significantly lower postoperative MD was observed in patients with optic tract contact with deformity (*P* = 0.009, **Figure 5F**).

Ultimately, the independent additional effects of nerve, tract and chiasm compression were assessed, with only Fujimoto grading being independently associated with preoperative MD (β = −2.43 (95%CI −3.323 to −1.54), P <0.001). Postoperatively, none of the compression locations were significantly associated with MD.

## Discussion

In this study, we investigated the association between radiological compression characteristics and visual field assessments, both by experts and using MD. When relating expert interpretation with MD, we found a highly sensitive cutoff value of −1.41 dB for affected vs. non-affected visual fields, and Δ2.52 dB for postoperative improvement in visual fields. Severity of optic chiasm compression, reflected by Fujimoto grades, correlated strongly with preoperative VFD, both for MD and for expert interpretation. This association disappeared for Fujimoto grades 1–3 after surgery, with only patients without optic chiasm contact (Fujimoto 0) and those with the highest grade of compression and deformity (Fujimoto 4) significantly differing in ophthalmological outcome.

Typically, NFAs compress the crossing fibers in the optic chiasm and cause pathognomonic bitemporal hemianopia. However, they can also cause quadrantanopia or atypical VFDs (10, 30, 31). In the present cohort, 47% of patients had typical hemi- or quadrantanopia, and 23% showed an atypical pattern, which may be explained by tumor location. However, additional compression of the optic tract and nerve still showed mainly temporal hemi- or quadrantanopia, suggesting that this type of radiological compression is not an obvious explanation for atypical VFD. We therefore hypothesize that other factors, such as vascular supply and the duration and onset of compression, may predict VFD patterns (32–34).

The degree and type of compression of the optic chiasm (i.e., Fujimoto grade), resulting from the suprasellar extension of the tumor, were associated with preoperative MD, as expected and reported by others (13–15, 35). Although large tumor volume (35) and an upwardly stretched and thinned optic chiasm (36) have been associated with poor visual improvement, preoperative MD may be more indicative of VFD recovery, as was observed in our cohort (data not shown) (16, 20–23).

In patients in whom NFMA was in contact with the prechiasmal optic nerve or optic tract, a trend toward lower preoperative MD was observed, which was in line with the findings of the Fujimoto grading. Postoperatively, the independent effects of all compression locations disappeared, indicating no impact on VFD recovery. Mechanistically, contact without deformity is hypothesized to be associated with worse visual function, potentially reflecting rapid increases in adenoma volume. The faster the compression occurs, the less time there is for adaptation, and therefore less chance for deformity. Anatomical deformity may therefore indicate greater adaptation to the altered anatomical situation, resulting in better visual function, although this requires further investigation.

According to Watanabe et al. (37), a hyperintense optic nerve on MRI was related to the persistence of postoperative visual impairment. Although the vast majority of our patients showed no hyperintensity or atrophy of the optic pathways, hampering analyses of this potential predictive factor, hyperintensity of the optic system did not exclude postoperative visual field improvement or normalization. However, the combination of atrophy and hyperintensity resulted in further deterioration after surgery in most patients and never in normalization of the visual fields. These results are in line with those of Zhang et al. (38), who correlated low preoperative chiasmatic volume with poorer visual prognosis at six months. Atrophy of the optic nervous system could provide greater predictive value for visual improvement.

In the last two decades, optical coherence tomography (OCT) techniques—to visualize (atrophy of) separate tissue layers of the macula and (peri)papillary regions—have been employed in the clinical ophthalmology (39, 40). OCT allows the deduction of the volume and mass of individual layers, and substantial evidence has accumulated that reduced mass of macular and papillary layers hampers visual recovery after chiasmic decompression surgery (22, 23, 41–43). Therefore, OCT may contribute to a more reliable visual prognosis compared with MRI and should be explored further in future studies (32, 34, 44–46).

Based on expert evaluation, 76% (n = 96) of the eyes with initial VFD showed postoperative improvement. Indeed, MD values improved significantly following surgery, in line with previous reports (7–9). However, 8% (n = 11) of the eyes showed deterioration: seven out of 11 had preoperative baseline VFD, and four of the 11 without preoperative VFD developed postoperative VFD. According to a meta-analysis by Muskens et al. (3), complete recovery occurs in only 30%–40% of cases, and 4% of patients experience deterioration of visual symptoms. Since our experts were masked to MD, their evaluation was solely based on visual field patterns, which could explain the higher incidence of VFD deterioration (3). VFD worsening may not be exclusively represented by lower MD but could depend on additional factors, such as the pattern of defect. Although expert reproducibility had near-perfect scores (ICC values of 0.821–0.926), MD reporting remains important to define the degree of VFD improvement. Therefore, establishing consensus MD cutoff values for VFD, as well as thresholds for dB improvement or worsening, is important.

Previously, studies have used different MD cutoff values to describe changes in VFD. Baseline VFD has been reported as <−5.0 dB (43), <−4.0 dB (20), <−3.0 dB (22), and <−2.0 dB (41), whereas “complete recovery” has been defined as >−2.0 dB (41) or ≥–3.5 dB (42), and “significant improvement” or “significant deterioration” as a change of ≥2 dB from the preoperative baseline MD (23). Using a >2 dB decrease from baseline MD to describe “deterioration of visual symptoms,” only four of 145 eyes would have deteriorated, highlighting the arbitrariness of MD cutoff values and underestimation of VFD compared with expert-opinion. Based on our study combining MD with expert opinion (47), we suggest using an MD cutoff value of −1.41 dB for affected visual fields, combined with expert evaluation to ensure reliability. A cutoff of Δ2.52 dB can be cautiously used to indicate improvement, ideally in combination with expert evaluation for patients with Δ1.24 dB–Δ2.52 dB improvement.

Several limitations of this study should be acknowledged. Due to the retrospective nature, imaging protocols and the timing of assessments were not standardized, limiting data control. Functional data, such as VFI and PSD, were not the focus of this study and therefore not included as outcome measures. Because of the long inclusion period (15 years), MRIs were older, of lower quality, and missing for many patients. Moreover, patients with low quality MRI, severe VFDs, or urgent need for surgery (e.g., apoplexy) were excluded, resulting in underrepresentation of this patient category and limiting the generalizability of the results. Furthermore, the number of eyes per compression grade or location (e.g., optic tract) was small, limiting statistical power.

In conclusion, preoperative compression of the optic chiasm (according to Fujimoto grade) was associated with lower preoperative MD. This study shows that, besides the Fujimoto grade, detailed preoperative MRI adenoma assessment—although correlated with preoperative VFDs—does not provide additional insights for predicting long-term VFD outcomes or explaining atypical VFD at presentations in the present study population. Notably, less than 50% of patients had typical temporal hemianopia. Based on this study, the relevance of expert assessment of visual fields warrants highlighting in both pre- and postoperative settings, for reliability and clinical evaluation. For research purposes, an MD cutoff value of −1.41 dB for determining affected visual fields and a potential cutoff of Δ2.52 dB for postoperative improvement require validation in other cohorts. Classification of dubious postoperative cases (i.e., ΔMD between Δ1.24 dB and Δ2.52 dB) should be performed by experts to determine clinical improvement.

## Data Availability

The raw data supporting the conclusions of this article will be made available by the authors, without undue reservation.
